# Task of leadership for intercultural opening strategies in organizations in adult and continuing education

**DOI:** 10.1365/s42681-021-00027-4

**Published:** 2022-01-18

**Authors:** Sonja Muders, Andreas Martin

**Affiliations:** Leibniz Centre for Lifelong Learning, German Institute for Adult Education, Heinemannstr. 12-14, 53175 Bonn, Germany

**Keywords:** Intercultural opening strategy, Leadership, Organizations in adult and continuing education (OACEs), Adult education centers (AECs), Effects of migration on participation in continuing education

## Abstract

In view of the growing number of people with a migration background, continuing education organizations are faced with strategic tasks of intercultural opening. These tasks require leaders to initiate and expand integration-promoting teaching work, to promote the intercultural sensitization of the members of the organization, and to develop an intercultural culture. This is a wide issue and concerns leadership action regarding funding, regular and special course programs, training for staff and lecturers, intercultural training, etc. In this qualitative study, the types of intercultural openness adopted by the management of adult education centers were categorized into proactive, reflexive, ambivalent, and symbolic types of openness. Using the procedure of exploratory sequential design, hypotheses were tested with the aim of gaining information that would facilitate broader statements about the field of adult education providers. The quantitative distribution was analyzed using a data set called ‘wbmonitor’ and a regional data set.

**Supplementary Information:**

The online version contains supplementary material available at 10.1365/s42681-021-00027-4.

## Intercultural opening strategies and practices of leaders in organizations in adult and continuing education

Migration and refugee movement are currently increasing worldwide. After the regulation on the free movement of persons was introduced within the European Union (EU), a new wave of refugees came to Europe in 2015/2016. This number of refugees led to increased demand for integration courses in Germany (Huntemann and Reichart 2017). Leaders of *organizations in adult and continuing education* (OACEs) were faced with the challenge of increasing the number of integration courses and expanding their education courses to provide language courses and special offers for refugees. Even before the arrival of so many refugees, OACEs already faced the challenge of providing education for the migration community, which includes groups from different backgrounds, milieus, and generations.

After the first phase of starting to tackle the problem in 2016, in 2017, it was time for strategic reflections. Consequently, leaders invested in the further education of their staff. Leadership in OACEs cannot be described with concepts of popular leadership styles. Instead, the concept of educational leadership is appropriate to characterize the situation of leaders of OACEs. Educational leadership (Herbrechter and Schrader 2018: 307f.) focuses more on the idea of education and on the organizational context (Robak 2004) and is thus not shaped by business management alone (and its idea of efficiency and effectiveness). Because OACEs have different management tasks and leadership is influenced by different factors, than those that apply to commercial businesses. *Diversity management* targets the interests of private individuals through its economic dimension (business and market-orientated), while *intercultural openness* targets public interests with its social dimension of public responsibility. The *gender mainstreaming* literature shows that forms of leadership are shaped by social gender roles and the biographical experience of managers (Sauer-Schiffer 2000). OACEs can use interorganizational collaborations and networks to approach and recruit new participants (Tröster et al. 2018). A further task for OACEs is dealing with conflicts in the workplace (Niebuhr 2011). In this article, we refer mainly to the management task of strategic intercultural opening, which concerns the organizational context, the recruiting of staff, the further education of staff (i.e., intercultural training or team building), volunteer management, and interorganizational cooperation. The strategy of intercultural opening and advancement will be referred to in this article as the *intercultural strategy* (ICS).

We examined the level of organization within OACEs because this level has the highest variance (Ioannidou and Jenner 2021). The differentiation at the organizational level is related to the hope that the tasks of continuing education can be better realized in special organizational forms and with plural association with association plural and by applying scientifically specific norms and principles of action rather than non-differentiated structures and principles (Tippelt 2018: 95).

For our study, we used the exploratory sequential design introduced by Creswell and Plano Clark (2011: 86ff) because it has an integrative design. In the second section of this article, we present the results of our qualitative research, which are based on the data obtained from leaders of *adult education centers* (AECs) in 20 interviews conducted in 2017. The AECs were chosen because of their mission statement on intercultural opening (on their homepage) and their activities in this field. Therefore, there are certain limitations to the qualitative part of this study. In the third section of this article, hypotheses for quantitative research will be generated from insides of AECs to OACEs (Creswell and Plano Clark 2011: 88). Our results are based on a data set called ‘wbmonitor’ and a regional data set. By using this procedure of exploratory sequential design, we were able to test hypotheses that comprised broad statements concerning the field of all providers in adult education.

## Qualitative analysis: intercultural strategies in adult education centers

The intercultural opening of AECs is an important prerequisite for promoting the equal participation of people from different backgrounds in continuing education. The urgency of the intercultural opening of AECs became more apparent as a result of the refugee movement to Germany in 2015, but also as a result of the recognized need to tackle educational inequalities and discrimination on the grounds of ethnicity or gender.

**Fig. 1:**
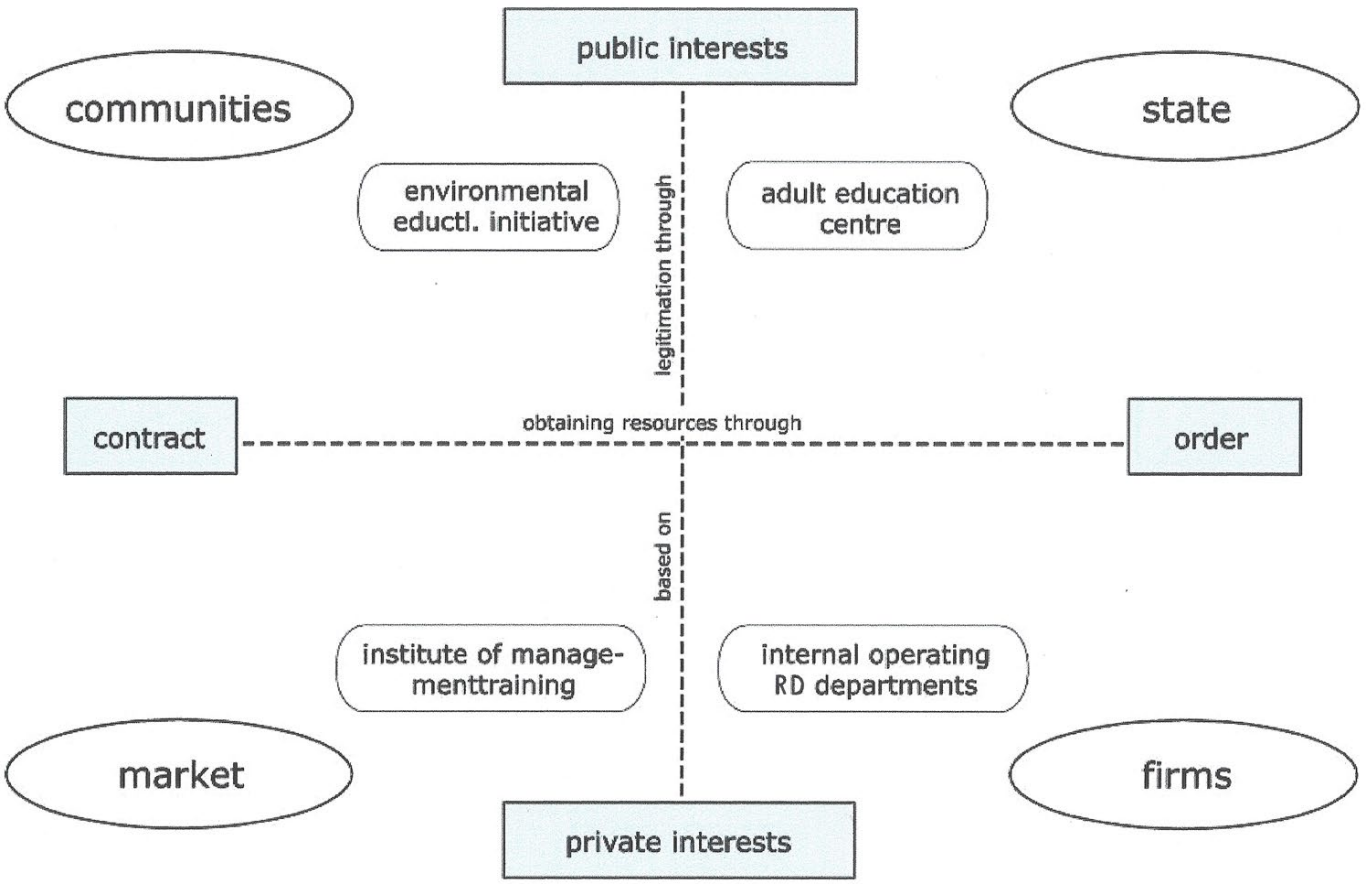
Reproduction contexts of continuing education (Schrader 2014: 60). Different providers are located in four different contexts: in the context of communities: environmental educational initiative, in context of state: adult education centre (AEC), in context of firms: internal operation RD (research and development) departments, in context of market: institute of management training

The approximately 900 AECs in Germany are located in the state sector. AECs are the largest provider of general adult education (594,000 courses with a peak of 17.9 million teaching hours, Huntemann and Reichart 2017: 18). The special role of AECs is based on the public order, which means that they have a public educational mandate that is regulated at different levels of the law (regional laws of the German federal states, national laws, and European law). AECs are justified through the public interest and maintained by public funding (see Fig. 1, reproduction contexts of continuing education—location of providers of Schrader (2014: 60). Therefore, AECs are supposed to be open to all people, regardless of age, disability, gender, origin, religion, worldview, social status, educational level, etc. (Süssmuth and Eisenfeld 2018: 764f). This means that their fees need to be socially affordable for all members of society and that reduced fees need to be offered to disadvantaged people. Therefore, due to this public mission of AECs, most AEC leaders aim to achieve intercultural openness.

To offer special services for specific target groups, knowledge about their quantitative distribution is required. The proportion of participants who have a migration background is not collected in the statistics of AECs due to their privacy policy. This is why a qualitative approach was chosen for this aspect of our study.

Intercultural opening represents an *organizational process* of change, in which the orientation of the educational institution can claim interculturality both internally and externally. Internal interculturality concerns principles of action, personnel structure, and the mission statement; external interculturality concerns target groups specific offers (Ruhland 2016). This leads to the following questions: How and to what extent does the intercultural opening of AECs succeed? What are the problems and challenges? What does intercultural opening mean for leadership? Which strategies are used? In what way do AECs, as educational organizations committed to the common good, react to migration-related social changes?

### Theoretical frame of reference: structuration and neo-institutionalism

The theoretical framework used for the qualitative part of this study was based on a structuration-theoretical understanding of organization. In this theory, an organization is seen as having a “reflexive structuration” (Ortmann et al. 1997: 315). With its focus on the practices and strategies of leadership, the theory explains that leadership actions both enable and limit action structures at the same time; this is called the “duality of structure” (Giddens 1988).

In addition, this study was based on the concept of neo-institutionalism. Basing the theoretical background on both structuration theory and neo-institutionalism seemed to be beneficial because both approaches share essential commonalities, and each approach can compensate for the central deficits of the respective other approach (Scott 1995; Barley and Tolbert 1997).

In the field of management research, organization-theoretical neo-institutionalism has become a central frame of reference (Schreyögg and Koch 2020: 60ff.). Its focus is on the relationship between management and the social structures of expectations. Neo-institutionalism is characterized by its view of organizations as being coupled with their environment. According to DiMaggio and Powell, organizations either voluntarily adopt orientation patterns (*normative isomorphism*), emulate pioneering models (*mimetic isomorphism*), or bow to external pressures (*coercive isomorphism*) (DiMagio and Powell 1983). So, adaptations lead institutionalized management practices to conform to expectations in order to gain legitimacy. Thus, this shows the importance of legitimacy attributions for organizations, which play the central role in the process of intercultural opening.

### Sample of adult education centers

A qualitative explanatory study was conducted because, due to data privacy regulations, statistics from AECs do not include data about the characteristics of participants, such as their migration background. The qualitative sample was chosen with the selection criterion of intercultural opening. The cases were chosen by experts in the field and double-checked by analyzing the AEC’s homepage with regard to their mission statement or a publicly visible commitment to intercultural opening.

With its *mission statement*, an organization communicates its self-image and its basic principles inside and outside the organization. Within the organization, the mission statement defines the orientation for action; outside the organization, it defines its public profile. The development of a mission statement is a management task. Ideally, the development of the mission statement is a joint process between management and employees, in which values are reflected upon. The participatory process is a cross-sectional task within the organization. The mission statement identifies the organization’s intercultural orientation as a sociopolitical mission.

An intercultural mission statement is made on the websites of 64% of AECs in Germany (Ambos et al. 2017: 34). Only this specific group, in which diversity is accepted at the organizational level, was interviewed in the qualitative study. Twenty AEC leaders were interviewed on three topics:

1. Situation and development since 2015 (wave of refugees): relevant developments in the characteristics of participants, especially participants with a migration background, in the range of programs offered, in the tasks for and demands on staff, and in cooperative relationships.
2. Special activities of AECs (Cooperation, course and program development, personell and organizational development, quality management).
3. Changes in strategic management.

Table 1 shows that 20 interviews were conducted with the leaders of six small-town AECs, 13 metropolitan AECs and one country-town AEC (AEC 4). AEC 4 is situated in an area with two towns and 22 communities, has a population of about 130,000, and is neighbored by AEC 16. The group of metropolitan AECs in our sample is larger because these AECs already started intercultural opening in the 1960s and thus have more experience in offering programs for migrants in the fields of German language courses, basic education, counseling, and support (Ruhland 2016: 28f.).

**Table 1:** Types of intercultural openness management, categorized into dimensions of cultural diversity based on dimensions of gender culture according to Funder et al. (2006: 203)

Cultural diversity	Proactive	Reflexive open	Ambivalent open	Symbolic open
Structural level				
Strategy	Intercultural further development and innovative	Intercultural further development, as intercultural opening is already taking place	Intercultural conflicts are resolved	Interculturality as an image: “AEC for all”
Expression/segregation/work allocation/resource allocation	Mixing of teams by employing staff with a migration background, egalitarian staffing at all hierarchical levels	Mixing of teams by employing staff with a migration background	Complementing the team by employing employees with a migration background, Women without migration background in leadership positions: unequal distribution of power and influence	Horizontal and vertical segregation No central decision-making positions with diversity category: unequal distribution of power and influence
Continuing education/training	Intercultural training for all	Need for action on intercultural training for all	Intercultural training or necessary coaching in case of conflicts	Intercultural training programs for the lecturers of integration courses
Symbolic-normative level				
Qualification, competences	Knowledge of role model function	Pragmatic view of subject matter and language skills, and awareness of positive impact of role modeling by members and lecturers	Expertise and cultural and language knowledge important	Qualifications/ expertise and degrees more important than cultural proximity to participants
Program area	Direct query of needs of the participants, Special course offers in English	Continuity and reaching out to communities, a lot of activities, Special offer for migrants	Focus on preservation rather than expansion of services	No special courses for migrants, no intercultural activities, open to all, emphasize that all are treated and addressed equally
Leadership style	Innovative and proactive, establishing a culture of diversity	Egalitarian and proactive	Thoughtful/ considered	Reactive
Examples of AEC	AEC 8 AEC 12	AEC 2 AEC 9 AEC 13 AEC 14 AEC 15 AEC 19 AEC 20	AEC 1 AEC 3 AEC 4 AEC 5 AEC 6 AEC 7 AEC 10 AEC 11 AEC 16 AEC 18	AEC 17

### Results on leadership tasks shaped by ICS

Leaders of publicly funded AECs face specific and new challenges due to the recent increase in migration. The expansion of integration courses requires great effort, because, for example, not only new courses but also new room capacities are needed. Intercultural opening is also accompanied by numerous conflicts and a shift to the language sector, “it was all about refugees and nothing else” (AEC 11). This shift brings lots of economic uncertainty, because certain requirements of the participants have to be presented for accounting purposes. In addition, new tasks occur for leaders because of the new increase in voluntary work and AECs’ involvement in this. So, volunteer management is a further new management task.

The special role of leaders in intercultural opening processes requires strategy development. Leaders must communicate and cannot really delegate intercultural openness, that is, they must set an example (in the sense of acting as a role model of intercultural openness) and work towards a positive response through understanding. The distinctive division of labor in AECs between management, planning, and teaching distinguishes between the functions of planning and teaching, which problematically fragments the pedagogical process and concerns the tasks of intercultural opening.

This leads to the research questions: How do AEC leaders deal with the new tasks? To what extent do leaders manage the intercultural opening of their AEC? How do AEC leaders develop an institution-specific strategy for intercultural opening?

Leaders of AECs must solve a different task every 20 min (f.e. AEC 3). So, leaders are constantly driven by time. This working environment is not an environment in which complex strategies can be easily developed. However, ICSs are chosen by numerous AEC leaders because of the demand for this special requirement of migration. Leaders must promote a mission statement, provide cross-team training and intercultural training for their staff, recruit staff with a migration background, and provide special offers for example. An ICS helps leaders with these tasks because it has solution strategies for several areas, including finance, contact with interorganizational *corporations*, finding new *staff* with intercultural competence (or providing further training for present staff), and the role of service (*consulting*). The successful implementation of these aspects can lead to higher participation rates.

Ultimately, leadership action is reliant on stakeholders, who are dependent on *funding*. Thus, AECs are characterized by the fact that they also have a public interest orientation and provide municipal public services. AECs’ funding is made up of public subsidies from the municipality, the city, state programs, and federal programs that can be further subdivided into funding from the Federal Office for Migration and Refugees (BAMF) and the Federal Employment Agency (BA). In the case of third-party funding, there are strict requirements from the federal government or the Federal Employment Agency, from EU programs, participants fee and/ or donations/sponsors.

Some leaders do not adopt an ICS because the entrepreneurial risk involved in the third-party funding required for German-as-a-second language (DAZ) courses is too high for them (e.g., AEC 4: 73). On the other hand, intercultural openness also has a positive economic component (AEC 16: 30): “we need these migrants as participants in the AEC because their participation also secures our existence in the long term. If we now have ever smaller social milieus coming to us, we will soon no longer be needed, and this is really an economic component of intercultural openness” (AEC 16: 6).

Depending on the legal form of the AEC, the leaders have greater or lesser degrees of freedom and more or less room for maneuver. Leaders of AECs manage different functional units of the organization, such as the administration and the teaching departments. The task of program planning is often delegated by leaders, so the staff in the departments makes the contracts with the lecturers. Thus, the *staff* (full-time employees) has an important role in intercultural opening processes (because the leaders relinquish planning responsibility to their staff). An explicit personnel recruitment strategy that focuses on recruiting full-time employees with a migration background and with intercultural competence is pursued only in a small proportion of AECs.

Leaders have to solve conflicts on different levels. Even if a commitment to the recognition of diversity is anchored in the AEC’s mission statement, this does not stop conflicts occurring that are related to migration-related diversity. Leaders are thus faced with the challenge of resolving conflicts. One main leadership task is to mediate *intercultural conflicts on different levels*. Leaders have to deal with conflicts caused by intercultural openness within the organization at the department level, conflicts with staff, conflicts with the cooperation partners that arise due to the participants or the lecturers in courses, and even conflicts about parking spaces.

The expansion of the departments responsible for language and integration in AECs can lead to increased internal competition between departments. This can, in turn, lead to conflicts located *inside the organization at the departmental level*. For example, in one of our interviews, the head of the German department stated: “I bring in the money here, nothing runs without me, we are the German department and in the German department everything runs differently” (AEC 10: 6). This led to a heated discussion because the other department heads did not feel valued and felt pushed aside. A coaching process had to be implemented to solve this bad working atmosphere (AEC 10: 5–7).

Conflicts even occur with *cooperation partners* due to participants. Some statements from our interviews suggested that the behavior of the refugee participants was not always considered acceptable. To tackle these problems, the German Adult Education Association provides rules for course participation in different languages such as Arabic and English. One AEC leader said: “We can then do talk to them that again and again and again, but it wears the tolerance down in the people who always encounter it.” (AEC 10: 9) This results in high fluctuation in the teaching staff as several employees leave AECs due to a certain level of exhaustion and the feeling that it is no longer possible for them to be open, friendly, or empathetic to the target group of participants (AEC 10: 9). Conflicts caused by intercultural opening were described as “the disreputable side” (by the leader of AEC 10: 9). Some leaders found solutions to this problem by providing further education training in languages such as Arabic and intercultural training courses, either in-house or externally, for their staff and lecturers. Other solutions were coaching (AEC 10) or implementation of structural changes (leader of AEC 20). The leader of AEC 20 changed the organization’s structure by creating a part-time position for a social worker whose job it was to clarify conflict cases, have the necessary discussions, deal with issues, and devote themselves specifically to the target group: “Until now, we have thought in terms of typical AEC structures, as in, there is a department, there is a department head, and then there are administrative employees, and that’s it. Now, at this point, you realize that the structure and the requirements of the department are changing” (AEC 20: 38). This example shows how an organization can be changed in order to react to requirements. Another example is the vocational orientation provided in AEC°18 for the participants of German language courses (AEC 18: 41).

Taking a closer look at changes in leadership, we found that leaders saw one of their main leadership tasks as foreseeing and catering for future developments. The leader of AEC 20 considered holding the organization together as a permanent development task:

*“(…) To be honest, I don’t think we’ll go back at all with regard to interculturality and diversity, but rather that this will continue and become even stronger. And, therefore, I believe that this is also a permanent development task for adult education centers, to handle this more and more professionally and to keep an eye on it. At the same time, however, and I think this is just as important, it is important to NOT lose sight of the fact that we are more than just a German language school, namely, an adult education center that consists of German and a WHOLE lot of other things and they are just as important, and it is important to hold the organization together. That is, I think, the overall task.” (AEC 20: 104).*

In 2017, disillusionment regarding intercultural opening set in among AEC leaders. Leaders had the problem that their AEC got an image problem if they mainly catered for migrants, refugees, and low-skilled people. Reconciling the different groups targeted by AECs, especially reconciling the target group of migrants and refugees with the educated middle-class clientele and advanced students, requires efforts. This brings us to the different types of ICSs.

### Types of intercultural openness management: proactive, reflexive, ambivalent, and symbolic

We categorized the statements obtained in our interviews according to Giddens’ heuristic of signification, domination, and legitimation. *Significations* rules regarding the constitution of meaning, which consider the guiding images and interpretative schemes in relation to cultural diversity. Authoritative-administrative and allocative resources are considered as *domination*, that is, the attribution of fields of activity as in the statement “they can’t drink that much tea with participants”, how course registrations were generated by staff with migration background. *Legitimation* concerns the rules of sanctioning action. Society’s normative ideas have changed. For AECs, a culture of diversity is nothing new; only society’s perception of it has changed:

*“So, these courses have been done since the founding of the adult education center, so it’s not something that’s really new, it’s just that now it’s perceived with completely different eyes, even the politicians perceive it with different eyes. For a long time, we were somehow an exotic association that did a bit of ikebana and other things somewhere, and now they are suddenly noticing how important the adult education centers have actually become and what an intercultural platform this also offers people.” (Leader of AEC 3: 49)*

A typology of dimensions/°strategies for intercultural opening looks at the strategy of the management concerning the corresponding leadership style and the action taken to promote intercultural development in the organizations. It looks at the culture of diversity and, if applicable, intercultural conflicts. These two perspectives—structuration theory and neo-institutionalism—complement each other and, thus, a typology on both a symbolic-normative level and a structural level is made possible.

A typology of gender cultures has already been operationalized by Funder et al. (2006: 193). This study followed that operationalization of the analytic dimension, as the concept of diversity management also includes aspects of gender and this operationalization captures the symbolic-normative level as well as the structural level of cultural diversity. This includes characteristics of horizontal/vertical segregation, forms of division of labor, and the distribution of power resources (Funder et al. 2006: 193).

When examining AECs as a place of cultural openness, the respective strategies of the AEC management can be classified as proactive, reflexive open, ambivalent open, or symbolic open. In this typology, we describe certain patterns in a condensed way. Therefore, the structural level is related to (1) strategies of intercultural opening/further development, qualifications, and competences, (2) areas of activity/program areas, and (3) leadership styles. In addition to this, this typology is also about the perception at the symbolic-normative level, which is related to (1) qualifications and competences, (2) program areas, and (3) leadership style.

To sum up: AECs have four types of cultural diversity; each type deals with ICS differently depending on the context. The six small-town AECs had all types, while the 13 metropolitan AECs were divided into two types: reflexive open and proactive. The proactive type is a rare lighting house case. The one country-town AEC was classified as the ambivalent type.

## Quantitative analysis of ICS in OACEs

Although ongoing transformations in the regional infrastructure of continuing education can be recognized, the need to change the direction of empirical research has become apparent. Following the mixed method exploratory sequential design (Creswell and Plano Clark 2011), we deduce hypotheses from the results of the qualitative study to test them. We assume an important role of context because of the difference of types of AECs. The purpose of using this design was to compare AECs’ decisions to implement an ICS with the decisions of other public and commercial training providers. We focused on the possible rationale behind AEC leaders’ decision to implement an ICS and on the effectiveness of such an ICS. The quantitative study pursued generalization. We thus conducted a quantitative investigation, in which we aimed to test three overall hypotheses about the ICS adopted by AECs compared with other OACEs.

### Hypothesis 1

ICS implementation as a function of funding and regional demand.

In the qualitative study, it became clear that all AECs were more or less engaged with an ICS. However, the form of the ICS and the extent to which it was implemented varied widely between AECs. These differences between AECs cannot be explained simply by the settlement structure and the number of migrants in the region. Therefore, in addition to these two aspects, the high engagement of AECs and their independence from regional demand, further aspects of the motivation of AECs compared to other education providers need to be investigated. According to Schrader (2010, 2011), it can be assumed that AECs tend to act normatively. In contrast to commercial providers, AECs do not orient themselves first and foremost to demand but to the guidelines and instructions of the policy that finances them. As intercultural openness is first and foremost a socio-political concept, we assumed that publicly funded providers such as AECs would implement an ICS to a greater extent than commercial providers. Because norms are always and everywhere equally binding, we did not expect to find any regional differences among normativ-rational actors. Thus, we expected that the likelihood of an ICS being adopted in publicly funded institutions, in contrast to commercial providers, would not increase as the proportion of migrants in the region increased. These assumptions were subdivided to form three hypotheses:

### Hypothesis 1a

The higher the proportion of public funding, the higher the probability of an ICS.

### Hypothesis 1b

The higher the proportion of migrants in the region, the higher the probability of an ICS.

### Hypothesis 1c

We expected to find a negative interaction effect between public funding and the proportion of migrants in a region.

### Hypothesis 2

ICS affects participation rates.

Hypotheses 1a, 1b, and 1c were based on the assumption that both commercial and non-commercial providers implement ICSs and that they intend (for different reasons) to increase the number of participants with a migration background. Also, in the qualitative study, most of the AEC leaders were of the opinion that it had not been possible to include people with a migration background in the regular program so far and that it was thus necessary to implement an ICS and adapt educational offers in this regard. It can be assumed that the consideration of migrants in the planning of the programs and courses relates to an increase in the number of participants because the expansion of the courses to target groups of participants who have not participated so far opens up additional participation potential. If this mechanism is effective, the effect of an ICS on participation rates could be expected to be higher in regions with a high proportion of migrants than in regions with a low proportion. This leads to two hypotheses that build on each other.

### Hypothesis 2a

An ICS increases participation rates.

This hypothesis assumed that the additional participation rates can be attributed to migrants. However, we did not have any direct information about the cultural background of participants in AECs. If an ICS indeed is connected to higher participation rates because additional migrants participate in continuing education, this means that the greater the proportion of migrants in the region, the better an ICS should work.

### Hypothesis 2b

The higher the proportion of migrants in a region, the stronger the effect of an ICS on participation rates.

### Hypothesis 3

Cooperation with migrant organizations.

Leaders of AECs reported that their main management tasks are strategic management and the coordination of collaborations, also called collaborative practices. One ICS is to cooperate with organizations in order to provide special offers for adults with a migration background (these types of cooperation are called interorganizational relationships).

So, an important aspect of an ICS is the cooperation between continuing education providers and migrant organizations. Migrant organizations act as gatekeepers to migrant communities. Cooperation programs with migrant organizations should therefore exploit this potential for participation, which can lead to more participants.

### Hypothesis 3a

Cooperation with migrant organizations increases participation rates.

Here, too, the hypothesis assumed that the additional participation rates would be due to migrants. However, we did not have any information about the cultural background of the participants. Therefore, we again used the proportion of migrants in the region to test this assumption.

### Hypothesis 3b

The higher the proportion of migrants in the population of a region, the stronger the effect of cooperation with migrant organizations on participation rates.

### Data analysis strategy

To test our hypotheses, we used data from the 2016 wbmonitor. The wbmonitor is an annual survey of continuing education providers in Germany conducted by the Federal Institute for Vocational Education and Training (BIBB) and the German Institute for Adult Education, Leibniz Centre for Lifelong Learning (DIE). It collects information on continuing education courses, participation, funding, personnel structure, and the perception of the economic situation.^1^ In addition, the wbmonitor collects information on a special topic every year. In 2016, this topic was “cultural diversity”. As part of this focus, the participating OACEs were asked about the extent to which they had adapted to the increased challenges posed by cultural diversity by adopting structural and organizational measures.

For the 2016 survey, 19,857 continuing education providers were contacted, and 1878 providers participated. These providers included all types of organizations whose primary business is the publicly accessible provision of continuing education. These are commercial providers, non-profit organizations, vocational schools, AECs, universities, organizations sponsored by churches, trade unions, and political parties, and business-related associations.

In Hypothesis 1, we tested the extent to which funding (1b) and regional context (1a) had an impact on the likelihood that an OAECs would implement an ICS. We assumed that publicly funded providers would be more likely to implement an ICS than other providers. To test this, we examined an interaction effect. If the expected effect was indeed due to the normative beliefs of public funders, the effect could be assumed to be independent of the actual need for intercultural openness. This need was defined by the proportion of migrants in the region. We operationalized ICS based on the question of whether the provider takes the development of migration into account in their program planning. This assumes that strategic orientation is best reflected in the actual courses offered. The most important independent variable is the degree of public funding. Here, we used a percentage to indicate the proportion of funding received from Europe, the federal government, the federal states, and the municipalities. To test the influence of the regional context, we used the population size of the spatial planning region (96 regions) and the proportion of migrants in the population. This information was obtained from the official statistics of the Federal Statistical Office.^2^ In addition, we controlled for the different provider types, the size of the provider (participation rates), and the population size and settlement structure of the region. A tabular list of all variables used can be found in the supplement.

To test Hypothesis 1, we used random intercept and random slope logit models. In the estimation, we allowed the constant and the effects of funding to vary across the spatial regions:

$${logP(y}_{ij}=1\left|{x}_{ij}\right)={\beta }_{0}+{\beta }_{1}{x}_{1j}+{(\beta }_{2}+{\zeta }_{2j}){x}_{2ij}+{\beta }_{3}{x}_{1j}{x}_{2ij}+\sum_{k=4}^{k=7}{{\beta }_{k}x}_{k}+{\zeta }_{1j}+{e}_{ij}$$

Here, ${x}_{1j}$ and ${x}_{2ij}$ are the independent variables migrant proportion of the population (this varied only at the regional level) and proportion of public funding in total funding. Variables ${x}_{4}$ to ${x}_{7}$ are the control variables, which varied either at the regional or the organizational level.${\zeta }_{1j}$ and ${\zeta }_{2j}$ are errors at the regional level.

In Hypothesis 2, we tested the influence of an ICS on participation rates. Our assumption here was that additional participation potential for people with a migration background can be exploited through intercultural openness. This could be assumed to increase the number of participants with a migration background in the courses of providers with an ICS. Because we were not able to directly measure the cultural background of the participants, we used the proportion of migrants in the region of the OACEs to test whether the additional participants were actually people with a migration background: if intercultural openness leads to more participants with a migration background, then, the higher the proportion of migrants in a region, the stronger the effect of an ICS on the number of participants should be.

To test Hypothesis 2, we used a random intercept model with random slope for the coefficient of intercultural openness. Again, the constant and the slope varied across the 96 spatial planning regions:

$${y}_{ij}={\beta }_{0}+{\beta }_{1}{x}_{1j}+{(\beta }_{2}+{\zeta }_{2j}){x}_{2ij}+{\beta }_{3}{x}_{1j}{x}_{2ij}+\sum_{k=4}^{k=7}{{\beta }_{k}x}_{k}+{\zeta }_{1j}+{e}_{ij}$$

The outcome here is the number of participants. ${x}_{1j}$ is the proportion of migrants in the total population in the region. This variable varied only between regions. ${x}_{2ij}$ is the binary variable for intercultural openness. ${x}_{4}$ to ${x}_{7}$ are the control variables (see Table S2 in the Supplement).${\zeta }_{1j}$ and ${\zeta }_{2j}$ are errors at the regional level.

In Hypothesis 3, we assumed that cooperation between AECs and migrant organizations would lead to higher participation rates. Migrant organizations act as gatekeepers and provide access to this specific population. The expected increase in participation could therefore be expected to be due to a higher number of participants with a migrant background. As we were not able to measure the cultural background of the participants, we again used the interaction with the proportion of migrants in the population to test whether the expected increase in participation could be explained by additional participation by people with a migration background.

To test Hypothesis 3, we used a random intercept model with a random slope for the coefficient of cooperation with migrant organizations. The constant and the slope varied across the 96 spatial planning regions:

$${y}_{ij}={\beta }_{0}+{\beta }_{1}{x}_{1j}+{(\beta }_{2}+{\zeta }_{2j}){x}_{2ij}+{\beta }_{3}{x}_{1j}{x}_{2ij}+\sum_{k=4}^{k=7}{{\beta }_{k}x}_{k}+{\zeta }_{1j}+{e}_{ij}$$

The outcome variable is again the number of participants. ${x}_{2ij}$ is the binary variable, cooperation with migrant organizations. ${x}_{1j}$ is the proportion of migrants in the population of the region. ${x}_{4}$ to ${x}_{7}$ are the control variables (see the Table S3 in the Supplement). ${\zeta }_{1j}$ and ${\zeta }_{2j}$ are the regional errors.

### Results of ICS implementation

Overall, the results show that our hypotheses were not confirmed for the most parts. In Hypothesis 1a, we had hypothesized that publicly funded providers would be more likely to implement an ICS. Because we assumed that public providers do this because it is politically required, we also assumed that this probability would not increase in line with the actual need for intercultural openness in the region. The results in Table 2 show that the likelihood of implementing a concrete ICS increased by nearly 1% when the proportion of public funding increased by 10%. The assumption that publicly funded providers are more likely to implement an ICS is further supported by the strong effect of AECs. The assumption formulated in Hypothesis 1b, which was that the higher the proportion of migrants in the region, the higher the probability of an ICS would be, was not confirmed. Instead, the probability decreased. However, this negative effect was smaller for publicly funded providers. Contrary to expectations (Hypothesis 1c), publicly funded providers paid more attention to the regional need for intercultural openness.

**Table 2:** Results for Hypotheses 1a, 1b, and 1c: ICS implementation as a function of funding and regional demand

ICS	Model 1		Model 2	
	Coef	*SE*	Coef	*SE*
Proportion of migrants	− 0.046*	0.021	− 0.069**	0.023
Proportion of public funding	0.009*	0.0023309	-0.002	0.005
Proportion of migrants*				
Proportion of public funding			0.001*	0.0004
Population size	1.75 × 10^–7^	1.02 × 10^–7^	1.80 × 10^–7^	9.97 × 10^–8^
Settlement structure (ref: Urban)				
With urbanization	0.069	0.181	0.067	0.177
Rural	− 0.098	0.230	− 0.085	0.227
Provider type (ref: Commercial)				
Non-profit	0.616**	0.179	0.600**	0.179
Company	0.195	0.329	0.197	0.329
Vocational school	− 0.121	0.281	− 0.142	0.278
Adult Education Center	0.913***	0.209	0.923***	0.208
Universities and colleges	− 0.250	0.352	− 0.276	0.352
Business associations	− 0.052	0.224	− 0.074	0.224
Churches, unions, pol. parties	0.256	0.181	0.250	0.181
Others (public)	− 0.341	0.445	− 0.438	0.445
Participation rates	10^–4^*	6.86 × 10^–6^	10^–4^*	6.88 × 10^–6^
Constant	− 0.600	0.310	− 0.326	0.3205509
Random part region				
*SD* constant	9.25 × 10^–11^		8.70 × 10^–8^	
*SD* proportion of public funding	0.004		0.001	

*p < 0.05; **p < 0.01; ***p < 0.001

It is also noteworthy that 94% of the regional variance in the effect of public funding was explained by the interaction with the proportion of migrants in the region.

In Hypothesis 2a, it was assumed that the implementation of an ICS relates to an increased participation rate. Although the results (see Table 3) show that an ICS had a positive effect, it was not significant. The assumption formulated in Hypothesis 2b that an ICS is connected to higher participation rates, especially in regions with a high proportion of migrants, was also not confirmed.

**Table 3:** Results for Hypotheses 2a and 2b: ICS affects participation rates

Participation Rates	Model 1		Model 2	
	Coef	*SE*	Coef	*SE*
ICS	1972.254	1033.838	− 296.095	2533.289
Proportion of migrants	240.232	132.806	204.434	137.708
Proportion of migrants*				
ICS			228.948	233.595
Population size	− 3 × 10^–4^	7 × 10^–4^	− 3 × 10^–4^	7 × 10^–4^
Proportion of public funding	8.746	12.550	8.445	12.551
Settlement structure (ref: Urban)				
With urbanization	587.030	1154.456	582.154	1153.787
Rural	28.729	1441.264	97.4905	1442.213
Provider type (ref: Commercial)				
Non-profit	− 21.352	1019.301	− 63.671	1019.944
Company	− 40.310	1838.882	− 74.623	1838.786
Vocational school	− 2083.32	1531.771	− 2120.576	1531.887
Adult Education Center	8829.02***	1139.476	8789.11***	1139.897
Universities and colleges	384.902	1833.292	362.090	1833.027
Business associations	1084.200	1205.107	1040.198	1205.646
Churches, unions, pol. parties	3996.182***	1017.486	3957.638***	1018.003
Others (public)	3149.58	2405.315	3074.898	2405.987
Constant	− 1952.213	1944.967	− 1583.732	1979.614
Random part region				
SD Constant	1.7 × 10^–6^		1.7 × 10^–6^	
SD ICS	7064.301		7037.644	

^*^p < 0.05; **p < 0.01; ***p < 0.001

Although the effect of an ICS on participation rates was not significant, it varied greatly across regions. Of this variance, only about 1% was explained by the proportion of migrants in the region.

In Hypothesis 3a, we assumed that cooperation with migrant organizations would lead to higher participation rates. Furthermore, we expected that the higher the proportion of migrants in the population of the region, the stronger this effect would be (Hypothesis 3b).

We indeed found a positive effect of cooperation with migrant organizations in Model 1 (see Table 4), but this effect did not increase when the proportion of migrants was higher (thus rejecting Hypothesis 3b). This suggests that the positive effect of the collaborations cannot be attributed to the higher numbers of participants from migrant communities.

**Table 4:** Results for Hypotheses 3a and 3b: Cooperation with migrant organizations

Participation Rates	Model 1		Model 2	
	Coef	*SE*	Coef	*SE*
Cooperation with migrant org	1990.215*	968.202	− 988.968	2309.45
Proportion of migrants	264.691	140.307	192.977	149.112
Proportion of migrants				
Cooperation with migrant org			296.090	208.816
Population size	− 3 × 10^–4^	7 × 10^–4^	− 3 × 10^–4^	7 × 10^–4^
Proportion of public funding	14.040	13.383	13.345	13.3851
Settlement structure (ref: Urban)				
With urbanization	748.826	1213.237	719.888	1210.344
Rural	86.282	1536.960	226.531	1536.392
Provider types (ref: Commercial)				
Non-profit	− 200.736	1071.278	− 287.080	1072.612
Company	− 205.913	2023.32	− 229.707	2022.723
Vocational school	− 2043.984	1631.183	− 2097.658	1631.095
Adult Education Center	8149.236***	1232.13	8048.248***	1233.787
Universities and colleges	375.383	1965.21	271.453	1965.866
Business associations	264.854	1269.466	190.561	1270.287
Churches, unions, pol. Parties	3972.512	1076.484	3878.407***	1078.418
Others (public)	2786.838	2485.935	2694.728	2486.149
Constant	− 2230.008	2077.379	− 1436.423	2147.230
Random part region				
SD Constant	3.6 × 10^–6^		1.1 × 10^–6^	
SD Cooperation with migrant org	5625.627		5535.101	

^*^p < 0.05; **p < 0.01; ***p < 0.001

The effect of collaborations also varied greatly between regions. Of this variance, only slightly more than 3% was explained by the proportion of migrants.

## Discussion: ICS in OACEs

The qualitative study showed that AECs are very committed to implementing ICSs. At the same time, it showed that they are not driven by the immediate needs of the regional context. From this, we derived the hypothesis that publicly funded AECs tend to act in a more normativ-rational way and differ from more commercial providers in this respect (see Hypotheses 1a, 1b, and 1c). Indeed, the results showed that, as the proportion of public funding increased, so did the likelihood of implementing an ICS. However, our results also showed that this does not happen independent of regional needs. In fact, the publicly funded institutions considered the regional needs to an even greater extent than the commercial providers did. It was observed that the overall proportion of migrants led to a decrease in the probability of an ICS implementation. One explanation for this unexpected result may be that commercial providers do not expect to benefit from a specific ICS as the proportion of migrants increases. Especially in large metropolitan cities with high migrant populations, the migrant community is expected to be very heterogeneous (ethnically, culturally, and socioeconomically). This fact targeted course programs and marketing strategies. Instead, strategies that appeal to a broad range of potential participants, regardless of their ethnic and cultural backgrounds, are much more effective. Publicly funded providers, on the other hand, seem to address migrants as a special target group to a greater extent, even though this group is very heterogeneous.

An ICS did not connect to higher participation rates, especially in regions with a high proportion of migrants. Hypotheses 2a and 2b were thus not confirmed. Also, the qualitative study ACEs leaders reported, they do not reach migrant communities in their regular program (AEC 3 etc.). A program plan oriented towards the market did not shape new developments; instead, society shaped new developments because “socially, a learning process has already occurred” (AEC 15: 99); this brought intercultural openness to publicly funded organizations as can explain by neo-institutionalism. However, the results show that the implementation of an ICS—as we operationalized it here—was not effective. A course program specifically tailored to migrants did not connect with additional participation. This did not work even when the migrant proportion in the population—that is, the target group—was very large. As publicly funded providers rely to a greater extent on such ICSs, this also strengthens the assumption that publicly funded providers, in particular, act normatively and that the actual effectiveness of an ICS does not play a central role.

Our discussion of the results for hypotheses 3a and 3b is based on the contradictory effects in models 1 and 2 in Table 4: although the results revealed a positive effect of cooperation with migrant organizations, this effect did not increase in line with a higher proportion of migrants (Hypothesis 3b). So, the positive effect of the collaboration was not attributed to higher participation rates from migrant communities. The literature suggests that the effect of cooperation leads to a higher participation rate in the following year (Martin and Muders 2018). However, for migrants as a heterogeneous group, the impact of cooperation with migrant organizations does not seem to be that strong.

The results of the exploratory interview study revealed that AEC leaders depending on the initial conditions tend to focus on sustaining or expanding the field of German courses (with external funding) in the organization. A public commitment to intercultural openness is not synonymous with 'best practice'. Leaders are faced with the challenge of resolving numerous intercultural conflicts at different levels. Depending on the nature of the conflict and the resources available, leaders choose the strategy of either using multilingual materials (such as rules for course participation in other languages) or providing intercultural training for their staff as in-house or external training (depending on resources). Coaching or hiring social workers were further individual strategies of certain AECs. So, most of the solutions by ICSs presented by leaders targeted individual rather than organizational processes and structures. The cultural change of the organization can hardly be generated by a single intervention such as a 1-day workshop on 'intercultural training' aimed at the behavior of individuals. This is also mentioned by Dobusch (2017). In her study, she focused on diversity discourse and discussed the criticism that diversity training focuses on the chance behavior of individual members instead of targeting fundamental change in the core principles of organizational inclusion policies. Therefore, change in individual instead of organizational processes and structures can be criticized.

The structuration theory can be used as an explanatory model, which shows that leadership actions and organizational structure reproduce each other, and the leadership actions performed in relation to intercultural opening significantly shape the OACEs.

The structuration approach is chosen because it overcomes objectivist and subjectivist explanations and furthermore because ICS are regulated social practices of leaders. Gidden’s heuristics of signification, domination, and legitimation were considered in the typing on page 6. But the structuration approach ignores the discrepancy between structural level and symbolic-normative level, while sociological neo-institutionalism focuses on structural level and symbolic-normative level. Thus, the combination of these two theories allows to explain patterns of change and stability of intercultural openness. These categories of analysis have been considered in Table 1. With neo-institutionalism, the rising number of AECs integration courses can be seen as a quick adaptation to the migration-related environmental changes. Which, moreover, still act economically, i.e. are oriented to their environment. So neo-institutionalism focused the relation of context of the organizations and the reaction of the organization specially to changing context like operationalized by an increasing migration quote in OACEs. Neo-institutionalism can provide an explanation of the good adaptation of ICS in AECs. Metropolitan AECs used to practice ICS for a long time and are divided into the two best practice types: reflexive open and proactive in the qualitative study. Adaption of ICS in AECs means on one hand focused on structural level: strategy, allocation, and training and on the other hand the symbolic-normative level: competences, program area and leadership style.

Mimetic isomorphisms—as we described at the beginning—can emulate pioneering models by (AEC 8, AEC 12). Also, the normative isomorphism can be used by metropolitan AECs here by adopt orientation patterns (AEC 2/9/13/14/15/19/20). By the way a mission statement by AECs does not mean best practice, as we see at the different types in the qualitative study. AEC leaders explain that mission statement is linked with a quality management tool and evaluation system, which was often introduce top down and not straight adopted in the culture (AEC 3/4/5/10/16). ACEs have been quick to adapt their course programs to the changing social context caused by migration, relocating study tours and in-company training to realize more capacity for language and integration courses. Against this background, the empirical findings can be understood as adaptation to the environment.

Our assumptions aim at causal relationships, but the data basis does not allow to test causal hypotheses to the full extent. In particular, the fact that we are dealing with cross-sectional data limits the possibilities of analysis. The conditions caused by the COVID-19 pandemic were not included in this study because the data on intercultural diversity did not allow for this. A future research topic will be the investigation of how learning formats for migrants changed as a result of the pandemic. Even though OACEs responded flexibly with online courses, face-to-face courses for migrants cannot be replaced.

Put simply, most AECs with an ICS see themselves as an organization open to everybody as opposed to an organization that just provides language-learning courses. This in turn influences the management tasks. The role of AECs is special in the field because of their public funding. So, our methodical design aims to a comparison of AECs with other OACEs and yielded generalization and verification of the results.

We know from our qualitative research that implementing an ICS as a leadership task takes a lot of time and energy but also offers many funding opportunities. We know from our quantitative study that the question of whether an AEC has an ICS is not directly related to the regional migrant proportion (in the municipality/city). The role of the AEC with its 'educational mission for all' comes into play more than expected, i.e. all citizens should continue to be addressed, not only refugees and language course participants. The combination of qualitative and quantitative methods shows that commercial and non-commercial organizations choose different strategies to deal with migration. Consequently, the results of the qualitative studies cannot be applied to all OACEs. Rather, they describe the situation in normatively oriented public continuing education organizations like AECs. Further research should examine whether these results will be affected in the context of digitization and whether regional educational inequalities can be reduced.

## Supplementary Information

Below is the link to the electronic supplementary material.

Supplementary file1 (DOCX 20 kb)

## Footnotes

1 For more information, see https://www.bibb.de/en/2160.php

2 Destatis: https://www.destatis.de/EN/Home/_node.html
