# Supplementary material for: Task of leadership for intercultural opening strategies in organizations in adult and continuing education
Source: Leadersh Educ Personal Interdiscip J. 2022 Jan 18;3(2):87–100. doi: 10.1365/s42681-021-00027-4 (PMC8764248; doi:10.1365/s42681-021-00027-4)
Supplement: Supplementary file 1 — Supplementary file1 (DOCX 20 kb) [file 42681_2021_27_MOESM1_ESM.docx]

Supplement

Table S1. Overview of the Adult Education Centers interviewed

Table S2. Summary of Variables in Hypotheses 1a, 1b, 1c, 2a, and 2 b

Table S3. Summary of Variables in Hypotheses 3a and 3b

Table S1

Overview of the Adult Education Centers interviewed

| Small AECs | Metropolitan AECs | Country AEC |
| --- | --- | --- |
| AEC 1  AEC 3  AEC 5  AEC 7  AEC 10  AEC 17 | AEC 2  AEC 6  AEC 8  AEC 9  AEC 11  AEC 12  AEC 13  AEC 14  AEC 15  AEC 16  AEC 18  AEC 19  AEC 20 | AEC 4 |

| Table S2  Summary of Variables in Hypotheses 1a, 1b, 1c, 2a, and 2 b | | | |  |
| --- | --- | --- | --- | --- |
| **Categorial Response** | ***M*** | ***SD*** | **Min/Max** | ***N*** |
| ICS | 0.42 | 0.49 | 0/1 | 1,403 |
| Provider types |  |  |  |  |
| Commercial | 0.24 | 0.43 | 0/1 | 1., 03 |
| Non-profit | 0.17 | 0.37 | 0/1 | 1,403 |
| Company | 0.03 | 0.18 | 0/1 | 1,403 |
| Vocational school | 0.07 | 0.26 | 0/1 | 1,403 |
| Business associations | 0.10 | 0.30 | 0/1 | 1,403 |
| Universities and colleges | 0.03 | 0.18 | 0/1 | 1,403 |
| Churches, unions, political parties | 0.18 | 0.39 | 0/1 | 1,403 |
| Adult Education Center | 0.16 | 0.36 | 0/1 | 1,403 |
| Others (public) | 0.02 | 0.14 | 0/1 | 1,403 |
| Settlement structure |  |  |  |  |
| Rural region | 0.20 | 0.40 | 0/1 | 1,403 |
| Regions with urbanization | 0.31 | 0.46 | 0/1 | 1,403 |
| Urban regions | 0.48 | 0.50 | 0/1 | 1,403 |
| **Continuous Response** | ***M*** | ***SD*** | **Min/Max** | ***N*** |
| Proportion of migrants in the region | 11.34 | 4.69 | 2.87/19.95 | 1,403 |
| Population size | 1,361,342 | 933,088 | 199,629/3,574,830 | 1,403 |
| Proportion of public funding | 24.95 | 30.81 | 0/100 | 1,403 |
| Participation numbers | 350,370 | 1,273,002 | 0/340,000 | 1,403 |
|  | | | |  |

| Table S3  Summary of Variables in Hypotheses 3a and 3b | | | |  |
| --- | --- | --- | --- | --- |
| **Categorial Response** | ***M*** | ***SD*** | **Min/Max** | ***N*** |
| Cooperation with Migrant Org. | 0.48 | 0.50 | 0/1 | 1,385 |
| Provider types |  |  |  |  |
| Commercial | 0.24 | 0.43 | 0/1 | 1,385 |
| Non-profit | 0.17 | 0.37 | 0/1 | 1,385 |
| Company | 0.03 | 0.17 | 0/1 | 1,385 |
| Vocational school | 0.07 | 0.25 | 0/1 | 1,385 |
| Business associations | 0.10 | 0.30 | 0/1 | 1,385 |
| Universities and colleges | 0.03 | 0.18 | 0/1 | 1,385 |
| Churches, unions, political parties | 0.18 | 0.39 | 0/1 | 1,385 |
| Adult Education Center | 0.15 | 0.36 | 0/1 | 1,385 |
| Others (public) | 0.02 | 0.14 | 0/1 | 1,385 |
| Settlement structure |  |  |  |  |
| Rural region | 0.20 | 0.40 | 0/1 | 1,385 |
| Regions with urbanization | 0.31 | 0.46 | 0/1 | 1,385 |
| Urban regions | 0.48 | 0.50 | 0/1 | 1,385 |
| **Continuous Response** | ***M*** | ***SD*** | **Min/Max** | ***N*** |
| Proportion of migrants in the region | 11.30 | 4.67 | 2.87/19.95 | 1,385 |
| Population size | 1,353,131 | 930,246 | 199,629/3,574,830 | 1,385 |
| Proportion of public funding | 25.01 | 30.71 | 0/100 | 1,385 |
| Participation numbers | 367,944 | 1,325,025 | 0/340,000 | 1,385 |
|  | | | |  |
